# Assessment of Practice and Outcome of Exercise on Quality of Life of Women with Breast Cancer in Delta State

**DOI:** 10.31557/APJCP.2021.22.8.2377

**Published:** 2021-08

**Authors:** Linda C Odikpo, Edith N Chiejina

**Affiliations:** *Department of Nursing Science, Nnamdi Azikiwe University Awka, Nnewi Campus, Nigeria. *

**Keywords:** Assessment, practice, outcome, exercise, generic quality of life

## Abstract

**Objectives:**

This study evaluated the effect of exercise on the quality of life of women with breast cancer. It determined the pre and post-intervention functional, symptoms, and global quality of life of women with breast cancer in the control and intervention groups.

**Methods:**

The quasi-experimental design study adopted a purposive sampling technique in selecting the women with breast cancer in the intervention (47) and control (47) groups. Data on QOL was measured using standardized instruments, namely the European Organization for Research and Treatment of Cancer Quality of Life Questionnaire-version 3 (EORTC QLQ-C30.

**Results:**

The results show the pre-intervention overall functional quality of life domain score was 59.2±21.98 for the intervention and 67.0± 20.13 for the control, an overall score for the symptom domain was 31.8±10.89 for the intervention and 29.8±10.24 for the control. The post-intervention overall generic functional domain score was 89.0±11.1 for the intervention and 51.2±17.8 for the control and for symptoms domain; their overall score was 16.4±10.2 for the intervention and 35.4±12.4 for the control. A significant difference existed in all post-intervention functional domain scores of the generic function and symptoms quality of life as the functional domains (p < 0.001) and global health status (p < 0.001).

**Conclusion:**

As the numbers of women who survive breast cancer continue to increase, there is a need for lifestyle modification like exercise to improve and maintain their overall QOL to live a fulfilled life devoid of post-treatment complications.

## Introduction

Breast cancer is the most common invasive cancer in women, and the second leading cause of cancer deaths in women, after lung cancer (American Cancer Society, 2013). Globally, breast cancer accounts for 45.1% of all cancers in women and the second leading cause of cancer death among women after lung cancer (Sathian et al., 2014). It is responsible for 23% of total cancer cases and 14% of cancer deaths worldwide (Jemal et al., 2011). 

Breast cancer is cancer that develops from breast tissue (National cancer institute, 2014). Breast cancers are considered either in situ or invasive (American Cancer Society, 2013). According to the Center for Disease Control (2017), the first symptoms of breast cancer is usually an area of thickened tissue in the breast or a lump in the breast or an armpit. Other symptoms of breast cancer include pain in the armpits or breast that does not change with the monthly cycle, pitting or redness of the skin of the breast like the skin of an orange, a rash around one of the nipples, a discharge from a nipple, possibly containing blood, an inverted nipple, a change in the size or shape of the breast, peeling, flaking and scaling of the skin on the breast or nipple.

Treatment for breast cancer depends on the type of breast cancer, the stage of cancer, sensitivity to hormones, the patient’s age, overall health, and preferences. The main treatment options for breast cancer, as outlined by American Institute for Cancer Research (2015), include surgery, radiation therapy, biological therapy, or targeted drug therapy, hormone therapy, and chemotherapy. This diagnostic and treatment process of breast cancer causes patients to have a more inferior quality of life which is negatively associated with the overall well-being of women with breast cancer (Sen et al., 2011; Yildiz et al., 2011; Enache, 2012). 

Studies on the quality of life of breast cancer patients show that poor health-related quality of life is always seen in breast cancer patients, especially in rehabilitation and aftercare. The focus is on reducing and improving cancer- and treatment-related side effects that do not subside even after the end of the therapy (Mirandola et al., 2014). Other symptoms associated with impaired quality of life include depression and fatigue, and quality of life is impaired not long after adjuvant treatment in breast cancer patients (Mirandola et al., 2014). Most of the problems associated with breast cancer diagnosis and treatment can be reduced or ameliorated with the help of exercise, as exercise has been seen to beneficial (Odikpo and Chiejina,2021). This view was supported by Mustian et al., (2017) in an overview of the oncology literature on the use of exercise to restore and improve the quality of life of cancer survivors. Breast cancer survivors in a study also indicated that exercise might have positive effects on their generic quality of life and disease-specific quality-of-life issues, including body image/self-esteem, emotional well-being, sexuality, sleep disturbance, social functioning, anxiety, fatigue, and pain (Vallance et al., 2013). There were also studies demonstrating that exercise was associated with improvements in the symptom of depression, maintenance of body weight, reduction of treatment side effects, body image, self-esteem (Volaklis et al.,2013; Odikpo and Chiejina,2021), and generic quality of life among breast cancer patients (Denlingerand Engstrom, 2011).

From the preceding, it is essential to note that reasonable evidence supports exercise interventions in breast cancer survivors have significant effects on their generic quality of life (QoL) by improving physical and psychological functioning, as discovered by Zeng et al., (2014) and Ferrer et al., (2011). Duncan et al., (2017) also stated that physical exercise interventions (yoga, aerobic, and resistance training) are effective in improving the generic quality of life in cancer survivors despite the prevailing barriers that prevent them from engaging in these health-promoting breast cancer-specific exercises (Odikpo and Chiejina, 2020).

In Nigeria, however, few studies have emerged on the quality of life of women with breast cancer, including a study by Adeoluwa et al., (2007) on health-related quality of life and its determinants in Nigerian breast cancer patients and Folorunsho et al., (2013) on quality of life of people with cancers in Ibadan, Nigeria. Despite the high incidence of breast cancer in Nigeria (Uchendu, 2020), none of these studies has considered exercise intervention and quality of life of breast cancer patients except study by Rufa’I et al., (2019) on Physical Activity and Health-Related Quality of Life in Breast Cancer Patients which was not an intervention study. Hence the need for this present study cannot be overemphasized as it aimed to assess the outcome of exercise on the generic quality of life of women with breast cancer in tertiary hospitals in Delta State, Nigeria. It determined the pre and post-intervention generic functional, symptoms, and global quality of life of women with breast cancer in the control and intervention groups. 

## Materials and Methods

The quasi-experimental design study adopted a purposive sampling technique in selecting the women with breast cancer in the intervention (47) and control (47) groups. It involved a pre-test, exercise intervention for the intervention group, and post-test data collection. The pre-test was carried out to collect baseline information on women’s quality of life with breast cancer in the intervention and control groups before exercise intervention was implemented for the intervention group. After the intervention, the post-test was conducted to assess the effect of exercise on women’s generic quality of life who participated in the study. The study area was two tertiary hospitals, including the Federal medical center, Asaba, and Delta State university teaching hospital Oghara that manage breast cancer patients in Delta state. The instrument for data collection in the study was divided into two sections; section A was used to elicit information on the demographic characteristics of the women in both groups, while section B was the European Organization for Research and Treatment of Cancer Quality of Life Questionnaire-version 3 [EORTC QLQ-C30] to elicit information on the pre and post-intervention quality of life of the women in both groups before and after the intervention. The generic QOL instrument comprised 30 items and was used to elicit information on the general health status of the respondents before and after exercise intervention. The QOL instrument is designed on a four-point scale with Not at all =1, Little =2, Quite a bit=3, and very much =4. Item 29 and 30 consists of a range of seven-point items (1234567) with extremes of poor =1 point and excellent =7 points. EORTC QLQ-C30 is a standardized instrument; hence, it was not validated but tested for reliability to ensure that it will adapt to our environment. The collected data were analyzed using the Spearman Brown formula for split-half coefficient. The reliability coefficient obtained was 0.85; hence the instrument was reliable for the study. The exercise intervention was conducted for the intervention group only with the assistance of the exercise physiotherapist, and the exercises practiced by the participants in the intervention group were warm-up with aerobic baseball and dance, treadmill, Ergometric bicycle riding, shoulder and arm exercises. After the exercise intervention, copies of the QOL questionnaire were re-administered to the two groups to collect post-test data. 

The summary of data collected was done using the average of the items that contribute to the scale of the QOL instrument, and hypotheses were tested using an independent sample t-test. The results were presented in Tables. The analysis was done using the Statistical Package for Social Sciences (SPSS) version 17.

**Table 1:** Socio-Demographic Characteristics of Participants

Variables	Intervention group (%)	Control group (%)	X^2^ value	p value
Age category				
Maximum age	64	63		
Minimum age	30	19	-	-
Mean ± SD	44 ± 8.0	45.0 ± 9.0		
Marital status				
Married	33 (70.2)	31 (66.0)		
Single	6 (12.8)	8 (17.0)	0.6	0.896
Widow	5 (10.6)	4 (8.5)		
Divorced/separated	3 (6.4)	4 (8.5)		
Level of education				
No formal education	3(6.4)	4 (8.5)		
Primary education	2(4.3)	6 (12.8)	2.7	0.448
Secondary education	15(31.9)	15 (31.9)		
Tertiary education	27(57.4)	22 (46.8)		
Religion				
Christian religion	42 (89.4)	45 (95.7)		
Islamic religion	5 (10.6)	1 (2.1)	3.8	0.152
Traditional religion	0 (0.0)	1 (2.1)		
Occupation				
Unemployed	0 (0.0)	6 (12.7)		
Self employed	14 (29.8)	17 (36.2)	7.7	0.021*
Employed	33 (70.2)	24 (51.1)		
Cancer diagnosis				
When were you diagnosed of breast cancer?				
ess than 3months	1 (2.1)	1 (2.1)		
3 to 6months	5 (10.9)	7 (14.9)		
7 to 9months	17 (37.0)	18 (38.3)	0.6	0.905
10 month and above	24 (52.2)	21 (44.7)		
At what stage were you diagnosed of breast cancer				
Stage1	27 (57.4)	20 (42.6)		
Stage 2	5 (10.6)	12 (25.5)		
Stage 3	1 (2.1)	0 (0.0)		
Stage 4	11 (23.4)	13 (27.7)	5.3	0.259
Not sure	3 (6.4)	2 (4.3)		

**Table 2:** Pre-Intervention Generic QOL of Women with Breast Cancer

Domain/Scales	Intervention M±SD	Control M±SD	Reference scores
Functional domain			
Physical functioning	55.8±21.6	58.5±19.9	≥ 66.7
Role functioning	47.2±22.3	56.3±27.2	≥ 50
Emotional functioning	60.2±19.8	65.6±19.5	≥ 50
Cognitive functioning	67.3±28.4	77.6±21.5	≥ 66.7
Social functioning	62.0±40.0	68.4±41.4	≥ 66.7
Overall functional score	59.2±21.98	67.0± 20.13	
Symptoms domain			
Fatigue	43.5±17.4	44.9±17.9	11.1-44.4
Nausea and vomiting	24.8±11.4	22.6±16.4	0-0
Pain	44.0±15.7	39.5±12.3	≤ 50
Dyspnoea	36.2±31.7	28.4±31.1	≤ 33.3
Insomnia	43.3±31.0	46.0±32.3	≤ 33.3
Appetite loss	6.3±14.9	17.0±27.6	≤ 33.3
Constipation	11.3±26.2	12.8±27.4	≤ 33.3
Diarrhea	14.0±28.4	3.5±12.4	0-0
Financial difficulties	61.7±33.3	53.9±35.8	≤ 33.3
Overall symptoms score	31.8±10.89	29.8±10.24	
Global health status/QoL (revised)	53.0±17.0	40.0±10.3	≥50

Physical func Q1-5, role func Q6,7, emotional func Q21-24, cognitive func Q20,25, social func Q26,27). Symptoms domain (fatigue Q10,12,18, nausea/vomiting Q14,15, pain Q9,19, dyspnoea Q8, insomnia Q11, appetite loss Q13, constipation Q16, diarrhea Q17, financial difficulties Q28).

**Table 3:** Practice of Exercise Observed by the Researcher for the Intervention Group of Women with Breast Cancer

Modality of exercises practiced	Number of weeks	Duration	Intensity	Notable side effect
Aerobic –dance, warm up with base ball, Resistance – threadmill and Ergometric bicycle riding flexibility- shoulder and arm exercises	<12weeks 14 (29.8) >12weeks 33 (70.2)	Aerobic 10-30munites 47(100) Resistance 10-20munites 47(100) Flexibility 10-20munites 47(100)	moderate 47(100)	Headache 3 (6.4) Dizziness 4 (8.5) Mild syncope 1 (2.1) None 39 (83.0)

**Table 4:** Post Intervention Generic QOL of Women with Breast Cancer

Domain/Scales	Intervention M±SD	Control M±SD	Reference score
Functional domain			
Physical functioning	89.1±15.3	45.4±24.3	≥66.7
Role functioning	88.7±15.2	57.1±25.9*	≥50
Emotional functioning	92.5±14.7	52.8±26.4*	≥50
Cognitive functioning	92.2±12.5	59.1±21.4*	≥66.7
Social functioning	82.6±23.0	41.5±27.9	≥66.7
Overall functional score	89.0±11.1	51.2±17.8	-
Symptoms domain			
Fatigue	13.5±15.2	44.9±17.8	11.1-44.4
Nausea and vomiting	6.0±13.6	32.9±28.9	0-0
Pain	12.4±14.9	42.6±22.7*	0-50
Dyspnoea	5.7±12.6	14.9±24.8*	0-33.3
Insomnia	15.5±27.7	48.2±22.8	0-33.3
Appetite loss	1.9±4.7	10.6±6.7*	0-33.3
Constipation	17.7±28.5	27.7±32.1*	0-33.3
Diarrhea	12.1±18.9	18.4±22.8	0-0
Financial difficulties	62.4±32.3	78.7±27.3	0.33.3
Overall symptoms score	16.4±10.2	35.4±12.4	-
Global health status/QoL (revised)	73.4±17.1	48.6±15.4	≥50

Physical functioning Q1-5, role functioning Q6,7, emotional functioning Q21-24, cognitive functioning Q20,25, social functioning Q26,27). Symptoms domain (fatigue Q10,12,18, nausea/vomiting Q14,15, pain Q9,19, dyspnoea Q8, insomnia Q11, appetite loss Q13, constipation Q16, diarrhea Q17, financial difficulties Q28).

**Table 5:** Independent Sample t-test of Difference in Generic Functional, Symptoms and Global Quality of Life of Women with Breast Cancer who Practiced and Those who Did Not

Domain/Scales	Intervention M±SD	Control M±SD	T	p-value
Functional domain				
Physical functioning	89.1±15.4	45.4±24.3	10.426	< 0.001
Role functioning	88.7±15.2	57.1±25.9	7.196	<0.001
Emotional functioning	92.6±14.8	52.8±26.4	9.009	< 0.001
Cognitive functioning	92.2±12.5	59.2±21.4	9.14	< 0.001
Social functioning	82.6±23.0	41.5±28.0	7.779	< 0.001
Overall functional score	89.0±11.1	51.2±17.8	12.353	< 0.001
Symptoms domain				
Fatigue	13.5±15.2	44.9±17.8	-9.217	< 0.001
Nausea and vomiting	6.0±13.6	33.0±29.0	-5.769	< 0.001
Pain	12.4±14.9	42.6±22.7	-7.596	<0 .001
Dyspnoea	5.7±12.7	14.9±24.9	-2.265	0.027
Insomnia	15.6±27.7	48.2±22.9	-6.232	< 0.001
Appetite loss	2.0±4.7	10.6±6.7	-7.268	< 0.001
Constipation	17.7±28.5	27.7±32.1	-1.585	0.116
Diarrhea	12.1±18.9	18.4±22.9	-1.474	0.144
Financial difficulties	62.4±32.3	78.7±27.3	-2.644	0.01
Overall symptoms score	16.4±10.2	35.4±12.4	-8.113	<0 .001
Global health status/QoL	73.4±17.1	48.6±15.4	7.404	< .001

**Table 6:** Independent Sample t-test of Comparison of Generic Functional, Symptoms and Global Quality of Life of Women in the Control and Intervention Group Pre-Intervention

Domain/scales	Intervention M±SD	Control M±SD	t	p-value
Functional domains				
Physical functioning	55.9±21.6	58.6±19.9	-0.628	0.531
Role functioning	47.2±22.3	56.4±27.3	-1.793	0.076
Emotional functioning	60.3±19.8	65.6±19.6	-1.31	0.194
Cognitive functioning	67.4±28.4	77.7±21.5	-1.978	0.051
Social functioning	62.1±40.0	68.4±41.4	-0.759	0.45
Overall functional score	58.6±18.8	65.3±17.0	-1.812	0.073
Symptoms domain				
Fatigue	43.5±17.5	44.9±17.9	-0.388	0.699
Nausea and vomiting	24.8±11.5	22.7±16.5	0.727	0.469
Pain	44.0±15.7	39.5±12.4	1.53	0.13
Dyspnoea	36.2±31.7	28.4±31.1	1.205	0.231
Insomnia	43.3±31.0	46.1±32.3	-0.435	0.665
Appetite loss	6.4±15.0	17.0±27.7	-2.317	0.023
Constipation	11.3±26.3	12.8±27.4	-0.256	0.798
Diarrhea	14.9±28.5	3.5±12.5	2.5	0.015
Financial difficulties	61.7±33.3	53.9±35.8	1.093	0.277
Overall symptoms score	31.8±10.9	29.8±10.2	0.918	0.361
Global health status/QoL	53.0±17.1	40.1±10.4	4.444	<.001

## Results

Table 1 shows that the minimum and maximum age of the respondents from Control group was 19years and 63years respectively, while that of the Intervention group was 30years and 64years. Both groups were mostly Christians [Intervention (89.4%); Control (95.7%)], and were married [intervention (70.2%); control (66.0%)]. Majority had tertiary education [intervention (57.4%), control (46.8%)], and were employed [intervention (70.2%); control(51.1%)]. Majority were diagnosed of breast cancer from 10 months and above for the both group [Intervention (52.2%); Control (44.7%)] and stages of which they were diagnosed was majorly stage 1 for the both groups [intervention (57.4%); control(42.6%)].

Table 2 above shows the generic functional quality of life of respondents from control and intervention group using EORTC instrument before intervention. For the control group, the functional, based on the reference points, values for role, emotional, cognitive, and social functions were found to be within the reference value at 56.3±27.2, 65.6±19.5, 77.6±21.5 and 68.4±41.4 respectively while physical functioning was low at 58.5±19.9. On symptoms, pain, dyspnoea, and, appetite loss and constipation were within the acceptable ranges at 39.5±12.3, 28.4±31.1, 17.0±27.6, and 12.8±27.4 respectively. However, symptoms such as nausea and vomiting, insomnia, diarrhoea and financial difficulties were quite high at 22.6±16.4, 46.0±32.3, 3.5±12.4 and 53.9±35.8 respectively. Meanwhile the global health status which shows the overall quality of life was low at 40.0±10.3. 

For intervention group, the functions, based on the reference points, values for emotional and cognitive, were found to be within the reference values at 60.2±19.8 and 67.3±28.4 respectively while physical, role and social functioning were low at 55.8±21.6, 47.2±22.3 and 62.0±40.0. 

On symptoms, fatigue, pain, dyspnoea, and, appetite loss and constipation were within the acceptable ranges at 43.5±17.4, 44.0±15.7, 6.3±14.9 and 11.3±26.2 respectively. However, symptoms such as nausea and vomiting, dyspnoea, insomnia, diarrhoea and financial difficulties were quite high at 24.8±11.4, 36.2±31.7, 43.3±31.0, 14.0±28.4 and 61.7±33.3 respectively. Meanwhile the global health status which show the overall QOL was fair at 53.0% although is few values above the border reference value. The overall functional quality of life was 59.2±21.98 for the intervention and 67.0± 20.13 for the control and overall score for the symptom domain was 31.8±10.89 for the intervention and 29.8±10.24 for the control. 

Table 3 shows the observed practice of exercise by women with breast cancer in the intervention group. Modality of exercises practiced was aerobic 47 (100), resistance 47 (100) and flexibility 47 (100). Majority 33 (70.2) practiced for a period of 12weeks,and time covered for aerobic 10-30 munites 47 (100), resistance 10-20 munites 47 (100), Flexibility 10-20 munites 47 (100). Intensity of exercise practiced mild and moderate 47 (100), majority did not have any side effects 39(83.0)

Table 4 presents post intervention generic functional symptom and global quality of life of women with breast cancer. In functional domain, emotional functioning (92.5±14.7), cognitive functioning (92.2±12.5), role functioning (88.7±15.2), physical (89.1±15.3) and social function (82.6±23.0) was higher for the intervention group of women than the control group, cognitive functioning (59.1±21.4), and role functioning (57.1±25.9), physical (45.4±24.3), emotional (52.8±26.4) and social functioning (41.5±27.9) respectively. In symptoms domain, all the symptoms were lesser for the intervention group post intervention than the control group. Global health quality of life for intervention was high and the improvement was remarkable at (73.4±17.1) and the control group scored (48.6±15.4). The overall generic functional quality of life was 89.0±11.1 for the intervention and 51.2±17.8 for the control and for symptoms domain, their overall score was 16.4±10.2 for the intervention and 35.4±12.4 for the control.

Table 5 revealed significant difference existed in all the functional domain of the generic quality of life as the intervention group had higher functional quality of life than the control group in all the functional domains (p < 0.001) and global health status (p < .001). Significant difference also existed in the symptoms quality of life as the control group had significantly higher symptoms than the intervention group except for constipation (p = 0.116) and diarrhea (p = 0.144), which were not significant.

Table 6 shows significance difference did not exist in all the functional domains of generic quality of life. For symptoms domain, significant difference existed only in appetite loss (p = 0.023) and diarrhoea (p = 0.015), with appetite loss being higher in the control and diarrhoea higher in the intervention. Significance difference also existed in global health status (p < 0.001).

## Discussion

Pre-intervention functional, symptoms and global status of the generic quality of life of women with breast cancer in the control and intervention groups shows the functional quality of life of the women with breast cancer in the control and intervention groups, apart from the low physical functioning, other functions were comparable to the reference values (Table 2). On symptoms, their symptoms were relatively high, and the overall global health status, which is the overall quality of life, was low for both groups of women. However, the intervention group had a score that is up to the minimal level of the reference point. This result on the quality of life considering the overall global health status/ QOL is poor, especially for women in the control group compared to the reference value of EORTC QOL. Hence, the overall QOL of women who participated in the study was inadequate for the control and fair for the intervention group, respectively. This finding is similar to Mirandola et al., (2014) and Yabroff et al., (2008), who stated that poor health-related quality of life is always seen in breast cancer patients, especially aftercare. However, a study by Muhammad et al., (2019) showed Global health status and functional scales, showing better QoL, although the symptoms were also on the high side, which is similar to that of the findings of this study. Also, a related study by Amarsheda and Bhise (2021) revealed that the breast cancer subscale score in QOL had a lower value than others, although the social well-being score was higher. 

The study also revealed high financial difficulty among the women in both groups. Corroborating this result, Breast cancer.org (2018) stated that breast Cancer Causes Long-Term Financial Burden for Many People, especially those With Lymphedema. Leez (2020) also stated that women with breast cancer battle with “Financial toxicity” — the cost-related side effects borne by cancer patients who take a huge emotional, mental and physical toll. Maggi (2020) and Spencer (2019) stressed that a similar financial burden said that women with breast cancer might likely change due to the illness, thereby posing more financial challenges. Jessica (2020) and Nahid et al., (2020) also reiterated that breast cancer survivors reported worsening financial status and distress after being diagnosed. There is, therefore, the need for emotional, financial, and physical support for these patients, and it is essential to state that with the current financial burden, all other aspects of generic QOL may be affected, thereby complicating the health of the women with breast cancer.

On the post-intervention generic functional, symptoms, and global quality of life of the control and intervention groups of women with breast cancer, for the control group, results show that most of the functional scales were low when placed side to side with the reference values. Most of the symptoms experienced were still high, and the global quality of life was still not up to the reference point. Hence, there was no remarkable improvement between the pretest and post-test results of the women in the control group who were not subjected to exercise intervention. On the post-intervention result of the intervention group regarding their generic functional, symptoms, and global quality of life, results show a remarkable improvement on all the domains of the generic QOL after the intervention. The exercise intervention improved the generic QOL of women with breast cancer in the intervention group, thereby agreeing with findings in a study by Margaret et al. (2015); David and Cynthia (2015). Hence, exercise had a beneficial effect on the generic QOL of life of breast cancer patients who participated in the study.

The hypotheses show a significant difference across all the functions, symptoms, and global health status between the two groups. For the functional domain and global health status, statistically significant improvement was observed across the scales for women who practiced exercise compared to those who did not. For the symptoms, a statistically significant decrease was observed in the values for women who practiced exercise compared to those who did not practice. Based on the preceding, the researcher rejected the hypothesis as it was evident that a lot of difference exists between the women from the intervention group who had the exercise intervention and the women from the control group who had none regarding all the domains of the generic QOL Domains. This finding was similar to report in a similar study by Ferrer et al., (2011). The result also supports evidence result that exercise interventions in breast cancer survivors have significant effects on generic quality of life(QoL) by improving the overall well-being of breast cancer patients (Zeng et al., 2014)

The pre-intervention hypotheses to show if a significant difference existed in the pre-intervention QOL of the participants in both groups revealed no significant difference in any of the functions between the women in the intervention and control groups pre-intervention. However, the researchers found few of the symptoms and global quality of life to differ within institutions, although the difference was not highly significant, thereby supporting evidence-based results by Muhammad et al., (2019) and Tahani et al., (2019). The latter saw similarity on issues that affect females with breast cancer. 

Based on the findings of this study, there is a need for urgent attention to be paid to the rehabilitation need of women with breast cancer, especially by those that care for them. Breast cancer patients should educate them on the need for exercise and its implication on their health primarily to ensure they have an improved and sustained generic QOL after their treatment. The exercise program should be specific and tailored to their need hence;, there is a need to use recommended exercise guidelines, which can be adapted to meet the local context and need of the individual breast cancer patient and other cancer patients in general. 

In conclusion, breast cancer has been a public health issue. As the numbers of women that survive breast cancer continue to increase, there is a need for lifestyle modification like exercise may improve and maintain the generic QOL of the sufferers so that they can live a fulfilled life devoid of post-treatment complications.

## Author Contribution Statement

All the authors contributing to the study from the beginning to the end of the study. Odikpo did manuscript drafting, while Chiejina supervised the process of the research till the end

## Data Availability

Data about the study is available with the corresponding author and will be provided upon reasonable request

## References

[B1] Adeoluwa J, Sofela EA, Adamu AR (2007). Health related quality of life and its determinants in Nigerian breast cancer patients. Afr J Med Health Sci.

[B2] Amarsheda S, Bhise A (2021). Association of fatigue, quality of life and functional capacity in breast cancer patients receiving adjuvant chemotherapy. Asian Pac J Cance Care.

[B3] American Cancer Society . (2013). Definition of breast cancer and cancer treatment. CA Cancer J Clin Med.

[B6] David OG, Cynthia AT (2015). Physical activity and cancer survivorship in the United States. Nutr Clin Pract.

[B7] Denlinger CS, Engstrom PF (2011). Cancer survivorship: movement matters. Cancer Prev Res.

[B8] Duncan M, Moschopoulou E, Herrington E (2017). SURECAN Investigators: Review of systematic reviews of non-pharmacological interventions to improve quality of life in cancer survivors. BMJ Open.

[B9] Enache RG (2012). The relationship between anxiety, depression and self-esteem in womenwith breast cancer after surgery. SBSPRO.

[B10] Ferrer RA, Huedo-Medina TB, Johnson BT, Ryan S, Pescatello LS (2011). Exercise interventions for cancer survivors: a meta-analysis of quality of life outcomes. Ann Behav Med.

[B11] Folorunsho N, Kazeem A, Olurotimi A (2013). Quality of life of people with cancers in Ibadan, Nigeria. J Ment Health.

[B12] Jemal A, Bray F, Center MM, Ferlay J, Ward E, Forman D (2011). Global cancer statistics. CA Cancer J Clin.

[B13] Jessica N, Semin D, Palm LM, Smith, Sarah R (2020). Understanding breast cancer survivors’ financial burden and distress after financial assistance. Support Care Cancer.

[B16] Margaret L (2015). Effects of exercise on breast cancer patients and survivors: a systematic review and meta-analysis. Curr Onco.

[B17] Mirandola D, Miccinesi G, Muraca MG (2014). Evidence for adapted physical activity as an effective intervention for upper limb mobility and quality of life in breast cancer survivors. J Phys Act Health.

[B18] Muhammad, Rolina, Shadi, Mukhtia, Bashayer (2019). Assessment of quality of life (QoL) in breast cancer patients: A tertiary care center survey in the western region of Saudi Arabia. PLoS One.

[B19] Mustian KM, Alfano CM, Heckler C (2017). Comparison of pharmaceutical, psychological, and exercise treatments for cancer-related fatigue: a meta-analysis. JAMA Oncol.

[B20] Nahid H, Vahid K, Asiyeh S, Hamidreza R (2014). The financial cost of preventive and curative programs for breast cancer: A Case Study of Women in Shiraz-Iran. Int J Health Policy Manag.

[B21] Odikpo Lc, Chiejina En 2020) Assessment of barriers to practice of exercise among women with breast cancer in Delta State. Breast J.

[B22] Odikpo Lc, Chiejina En (2021). Knowledge and perceived benefits of exercise among women with breast cancer in Tertiary Hospitals in Delta State. Breast Dis.

[B23] Rufa’i AA, V Muralikrishna BV, Yen SH, Wan-Muda WM (2019). Physical activity and health-related quality of life in breast cancer patients: A Multicenter Cross-Sectional Survey. Middle East J Rehabil Health Stud.

[B24] Sathian B, Nagaraja, SB, Banerjee I (2014). Awareness of breast cancer warning signs and screening methods among female residents of Pokhara valley, Nepal. Asian Pac J Cancer Prev.

[B25] Sen F, Aydiner A (2011). Depending on the late side effects of cancer treatment. J Clin Dev.

[B26] Spencer JC, Rotter JS, Eberth JM (2019). Employment changes following breast cancer diagnosis: the effects of race and place. J Natl Cancer Inst.

[B27] Tahani HN, Huda RE, Aisha OG, Arwa AO, Raid (2019). Quality of life assessment of breast cancer patients in Saudi Arabia. PLoS One.

[B28] Uchendu O (2020). Cancer incidence in Nigeria: A Tertiary Hospital Experience. Asian Pac J Cance Care.

[B29] Vallance JK, Courneya KS, Plotnikoff RC, Yasui Y, Mackey JR (2013). Randomized controlled trial of the effects of print materials and step pedometers on physical activity and quality of life in breast cancer survivors. J Clin Oncol.

[B30] Volaklis KA, Halle M, Tokmakidis SP (2013). Exercise in the prevention and rehabilitation of breast cancer. Wien Klin Wochenschr.

[B31] Yabroff KR, Lamont EB, Mariotto A (2008). Cost of care for elderly cancer patients in the United States. J Natl Cancer Inst.

[B32] Yildiz A, Karayurt O (2011). Women with breast cancer lymphedema due to the difficulties in which they live. Breast Health J.

[B33] Zeng YC, Huang ML, Cheng A, Zhou Y, So WK (2014). Meta-analysis of the effects of exercise intervention on quality of life in breast cancer survivors. J Breast Cancer.

